# A Novel Case of Citrobacter koseri Septic Arthritis in a Native Knee Joint

**DOI:** 10.7759/cureus.81066

**Published:** 2025-03-24

**Authors:** Sreeram Ravi, Kaitlin L Saloky, Rachel Thomas, Edward Fox, Meredith Schade

**Affiliations:** 1 Orthopedics, Penn State Health Milton S. Hershey Medical Center, Hershey, USA; 2 Orthopedics and Rehabilitation, Penn State Health Milton S. Hershey Medical Center, Hershey, USA; 3 Infectious Disease, Penn State Health Milton S. Hershey Medical Center, Hershey, USA

**Keywords:** knee joint infection, knee swelling, painful knee, septic arthritis, septic arthritis of knee

## Abstract

*Citrobacter koseri* is a rare cause of septic arthritis in a native knee joint. We present the first recorded case of *C. koseri* septic arthritis in the native knee from a hematogenous spread resulting from a gastrointestinal illness, along with a review of the literature. A search of PubMed, Embase, OVID, and Google Scholar was conducted. Fifteen cases of musculoskeletal infection were identified, and of these, only six cases involved a septic joint and one case involved a septic knee. None of the recorded adult cases presented hematogenously without prior trauma. All of the cases achieved resolution after either antibiotic treatment alone or in conjunction with surgical treatment. *Citrobacter koseri* is not a rare cause of infection in general. However, it is not a common cause of septic arthritis, and this may complicate the treatment course as it can be overlooked as a cause of infection. It is essential to obtain a thorough history and a broad differential diagnosis when evaluating septic arthritis as although it may be difficult to identify the infection, *C. koseri* septic arthritis can be effectively treated with surgery as well as newer generation beta-lactams and cephalosporin antibiotic therapy.

## Introduction

Septic arthritis is a serious cause of inflammatory arthritis, with an incidence of two to six cases per 100,000 and a prevalence that can vary based on age and comorbidities [1]. Septic arthritis is usually bacterial or monoarticular and, although uncommon, is an orthopedic emergency that, if untreated, can lead to permanent joint damage and increased morbidity and mortality. In an immunocompetent adult, the most common pathogen is *Staphylococcus aureus*; however, other bacteria such as *Neisseria gonorrhoeae*, *Salmonella* spp., and *Pseudomonas aeruginosa* have been described with various risk factors, including trauma, drug use, and sickle cell disease [2]. *Citrobacter koseri* is a non-sporulating, aerobic, gram-negative bacilli in the *Enterobacteriaceae* family that is a common cause of gastrointestinal illness and nosocomial illness that has not previously been shown to infect native joints in adults [3]. This report seeks to present the first case of septic arthritis caused by C. *koseri* of a native knee in an immunocompetent adult without antecedent trauma.

## Case presentation

The patient is a 54-year-old male with no significant medical history and no relevant surgical history who presented to the emergency department after two days of left knee pain and swelling with difficulty ambulating. He did note a gastrointestinal illness and sore throat four days prior to presentation but denied any recent trauma or any history of surgery to the knee. On examination, his knee was grossly swollen, with notable effusion, and non-erythematous, and range of motion was 0-80 degrees, with no pain and a short-arc range of motion. His blood laboratory values on presentation were as follows: white blood cell count was 20.1K/uL, erythrocyte sedimentation rate (ESR) was 16mm/hr, and C-reactive protein (CRP) was 32.4mg/dL. Arthrocentesis was performed with manual cell count and differential, which demonstrated 20,718 cells, 87% neutrophils, and no crystals (Table 1). Synovial fluid cultures and Lyme PCR were sent to the lab and results were in process. Based on his equivocal examination and low cell count in the synovial fluid, the patient was discharged from the hospital with close follow-up. He was not discharged on any antibiotics due to low concern for septic arthritis. The following day, the synovial fluid culture grew *C. koseri*, and the patient was brought back to the hospital for irrigation and debridement of his culture-positive septic arthritis. Upon presentation to the hospital, he had increased effusion of the left knee, increasing knee pain, and worsening difficulty with ambulation. His blood laboratory values on presentation were as follows white blood cell count was 13.45K/uL, ESR was 34mm/hr, and CRP was 17.43mg/dL. The patient was taken to the operating room for irrigation and debridement of his left knee, and there was an uneventful operative course, with an open arthrotomy, along with thorough irrigation, debridement, and drain placement. Intraoperative tissue and fluid cultures were taken and sent for analysis. The patient’s blood, tissue, and intraoperative fluid cultures grew *C. koseri*. The patient was started on intravenous cefepime and transitioned to ceftriaxone for a four-week course upon discharge.

**Table 1:** Patient's laboratory values WBC, white blood cell; ESR, erythrocyte sedimentation rate; CRP, C-reactive protein Reference values as established at Penn State Milton S. Hershey Medical Center

Lab	Value	Normal value	Source
WBC	13.45K/uL	4K/uL-10K/uL	Serum
ESR	34mm/hr	0mm/hr-25mm/hr	Serum
CRP	17.43mg/dL	<0.5mg/dL	Serum
Cell count	20,718 cells/uL	<200 cells/uL	Synovial fluid
Neutrophil	87%	<20%	Synovial fluid

**Figure 1:**
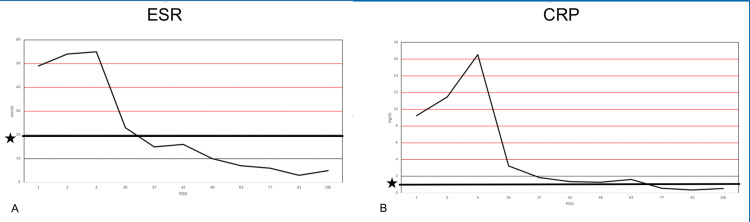
Inflammatory marker trend (A) ESR trend line over follow-up interval, with the bolded black line denoted with a "star" representing the institutional normal reference value. The Y-axis represents ESR value in mm/hr, and the X-axis represents POD (postoperative day). (B) CRP trend line over follow-up interval with bolded black line denoted with a "star" representing the institutional normal reference value. The Y-axis represents CRP value in mg/dL, and the X-axis represents POD (postoperative day). ESR, erythrocyte sedimentation rate; CRP, C-reactive protein

**Figure 2:**
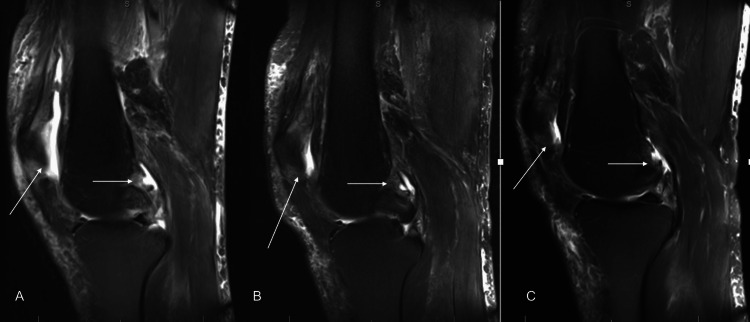
Serial postoperative MRI MRI taken at six weeks postoperatively (A), six months postoperatively (B), and one year postoperatively (C). The arrows represent areas of effusion and the anterior knee arrow also points to anterior synovitis.

At his four-week postoperative visit, the patient showed rapid improvement with improved range of motion and no pain with ambulation. He continued to have small knee effusions with a healed surgical incision, and he was transitioned to oral cephalexin for 10 days. He was closely followed postoperatively, and during his postoperative course, serial inflammatory markers were obtained, which trended until he had close to normal values, which occurred around his postoperative day 77 visit (Table 2, Figure 1). MRI was obtained at week 6, month 6, and one year postoperatively, which showed a progressive decrease in anterior synovitis and joint effusion (Figure 2). Due to continued clinical and laboratory improvement, after his one-year postoperative visit, he was discharged from follow-up.

**Table 2:** Inflammatory marker trend Reference values are at the top of each column. Values trend downwards at each subsequent follow-up visit, with the final visits prior to discharge from follow-up having normal or marginally above normal values.

Reference values	0mm/hr-20mm/hr	<0.5mg/dL
Time	ESR (mm/hr)	CRP (mg/dL)
POD 1	49	9.23
POD 2	54	11.46
POD 3	55	16.54
POD 30	23	3.22
POD 37	15	1.85
POD 42	16	1.36
POD 48	10	1.27
POD 63	7	1.60
POD 77	6	0.57
POD 91	3	0.34
POD 105	5	0.52

## Discussion

Septic arthritis is commonly caused by bacteria such as *Staphylococcus aureus*, *Streptococcus* spp., and *Neisseria gonorrhoeae*. In most cases of native and prosthetic joint infections, patients present with classic laboratory and physical examination findings. In this report, we omit discussion of prosthetic joint infections [2]. The laboratory findings include serum WBC count of greater than 10K cells/mL with left shift, ESR greater than 30mm/hr, CRP greater than 1mg/dL, with fluid aspirate results of WBC count greater than 50,000 cells/mL [2-4]. Physical examination findings include pain in the affected joint, fevers, effusion, and erythema. His knee was held in slight flexion, which showed an inability to bear weight and tolerate a passive range of motion.

Diagnosis of septic arthritis is an important part of the general skill set, as early diagnosis can lead to early operative or pharmacologic treatment. Time to treatment is important as prolonged infection can lead to cartilaginous destruction as well as hematogenous spread and bacteremia, which can result in sepsis [2]. This case of *C. koseri *septic arthritis is notable because the patient was initially discharged, as his clinical presentation did not correlate strongly with a classic presentation of septic arthritis. The patient’s initial history, including antecedent gastrointestinal illness involving fevers, chills, and abdominal pain, as well as the positive fluid culture for *C. koseri*, provided a high index of suspicion for active infection [3,5].

*Citrobacter koseri* is a non-sporulating, aerobic, gram-negative bacilli in the Enterobacteriaceae family. The majority of infections from the genus Citrobacter are from *C. freundii* and *C. koseri* and are frequently found in soil, water, and the normal gastrointestinal and genitourinary tract of many mammals, including humans. Citrobacter infections have almost exclusively been found to cause central nervous system infections in neonates and gastrointestinal or genitourinary infections in elderly and/or immunocompromised individuals. They are overwhelmingly nosocomial infections, with more than 70% being hospital-acquired or the result of invasive procedures [3,6-18].

Our case is a unique one as all prior instances of septic arthritis of major joints in the literature involved prior trauma to the joint either via fracture or recent antecedent surgery, as shown in Table 3. This case involved a native joint with no prior surgery or antecedent trauma. The most similar relevant case is described by Kwaees et al. [3], which describes an elderly patient with a medical history significant for gout and who had recently undergone arthroscopy of the affected knee. This differs from our case involving a patient with no known chronic conditions and no trauma to the infected knee. The only predisposition to this infection this patient had was a gastrointestinal illness and cough one week before infection. The only other cases in the literature on *C. Koseri* septic arthritis innative joints involved the sternoclavicular joint, shoulder joint, and the hip joint, and all of the other cases involved prior surgery or trauma [6,7,9-11].

**Table 3:** Cases of Citrobacter koseri septic arthritis of the knee This table includes prior case reports of *Citrobacter koseri *septic arthritis, as well as the patient age, affected joint, and any relevant risk factors.

Author	Year	Age	Site	Native	Risk factor
Kwaees et al. [3]	2016	72	Knee	Yes	Arthroscopy, gout, hypercholesterolemia
Holt et al. [6]	1981	47	Sternoclavicular joint	Yes	Hypoprothrombinemia, alcoholism
Fuxench-Chiesa, et al. [7]	1983	45	Sternoclavicular joint	Yes	Alcoholism
Hayani [9]	2011	Neonate	Shoulder	Yes	Fracture
Maynou et al. [10]	2006	54	Shoulder	No	None
Kaufman et al. [11]	2011	53	Hip	No	Diabetes, gout, obesity, hypertension
Present case	2023	54	Knee	Yes	None

## Conclusions

*Citrobacter koseri* is an aggressive but uncommon cause of musculoskeletal infection; thus, it may not be high on a clinician’s differential diagnosis. Moreover, patients may have atypical presentations that may elude the criteria for aggressive treatment. These factors, in conjunction, can potentially lead to a delay in treatment and, ultimately, cartilaginous destruction. This case is unique in that the patient was a relatively healthy 54-year-old male with no immunocompromise, recent trauma, antecedent surgery, or infection of the affected joint. All of the prior reported cases involved these risk factors. Therefore, in adult immunocompetent patients with atypical presentation that is not overtly concerning for septic arthritis, it is important to recognize *C. koseri *as a potential cause of infection.
